# ZWZ-3, a Fluorescent Probe Targeting Mitochondria for Melanoma Imaging and Therapy

**DOI:** 10.3389/fphar.2022.829684

**Published:** 2022-02-23

**Authors:** Zengjin Liu, Hailan Wang, Changzhen Sun, Yuanmin He, Tong Xia, Jianv Wang, Xia Xiong, Qingbi Zhang, Sijin Yang, Li Liu

**Affiliations:** ^1^ National Traditional Chinese Medicine Clinical Research Base and Drug Research Center of the Affiliated Traditional Chinese Medicine Hospital of Southwest Medical University, Luzhou, China; ^2^ Department of Dermatology, The Affiliated Hospital of Southwest Medical University, Luzhou, China; ^3^ School of Public Health, Southwest Medical University, Luzhou, China

**Keywords:** hemicyanine-based fluorescent probe, anti-cancer, mitochondrial-targeted, autophagy, apoptosis

## Abstract

The increased drug resistance and metastasis of melanoma resulted in poor prognosis of patients. Here, we designed and synthesized a novel hemicyanine-based fluorescent probe ZWZ-3, and investigated its application for melanoma imaging and treatment both *in vitro* and *in vivo*. ZWZ-3 preferentially accumulated in melanoma cells *via* a process that depended on the organic anion-transporting polypeptide (OATP), which targeted mitochondria on the hemicyanine cationic nitrogen. In addition, we investigated the effect and molecular mechanism of ZWZ-3 in melanoma. *In vitro* studies showed that ZWZ-3 promoted the generation of reactive oxygen species and induced mitochondrial-mediated cell apoptosis by upregulating Bax and activating caspase-3, caspase-9, and PARP. Importantly, ZWZ-3 also induced autophagy by upregulating LC-3II and Atg5 and downregulating P62. It significantly suppressed tumor growth of A375 xenograft tumor in mice without notable side effects. Histological and immunohistochemical analyses revealed that ZWZ-3 induced apoptosis and inhibited tumor cell proliferation. Thus, ZWZ-3 represents a novel theranostic agent that can be used to effectively targeting, detecting, and treating melanoma. It could also help monitoring disease progression and response to treatment.

## Introduction

Melanoma is one of the most malignant tumors with high metastatic potential. Although it represents only 4% of dermal tumor cases, melanoma is the most dangerous and deadly form of dermal tumor, accounting for 75% of skin cancer-related deaths (Apalla et al., 2017; Hessler et al., 2020). Early-stage melanoma can be treated with surgical intervention, with relatively high 5-year survival rate, yet metastatic melanoma significantly shorten survival rate (Smart et al., 2021). Due to its aggressiveness, melanoma patients are often diagnosed at advanced stages with local infiltration or distant metastasis, which cannot be treated with surgery alone (Tse et al., 2017; Zhu et al., 2017). In addition, melanoma is one of the most potent drug-resistant cancers (Wang et al., 2015). Thus, multifunctional theranostic agents with the potential of simultaneous imaging-guided, tumor targeting and treatment are urgently needed to improve the timely diagnosis and treatment of the disease.

Cancer cell death involves various pathways including apoptosis, necrosis and autophagy. Apoptosis is a type of programmed cell death in which reactive oxygen species (ROS) are abnormally generated in response to diverse external stimuli, which enhanced oxidative stress in mitochondria and dysregulated the mitochondrial membrane and proteins (Cotter, 2009; D’Arcy, 2019). The released mitochondrial protein cytochrome C causes activation of caspase-like proteases, and leading to apoptotic cell death (Bajpai et al., 2020). Autophagy plays an important role of “garbage collector”, which removed damaged cellular components or abnormal metabolites. During the process of autophagy, autophagosomes engulf cytoplasmic components, during which the cytosolic form of LC3, called LC3-I, converts into the autophagosome-associated LC3-II form (Huang et al., 2013; Zada et al., 2021). This conversion can therefore be used for tracking autophagy, as can the interaction between LC3 and the adapter protein p62 during autophagosome formation (Islam et al., 2018). The adapter protein P62 also interacts with polyubiquitinated proteins through the ubiquitin-associated domain to promote the autophagic degradation of ubiquitinated substrates (Zhang et al., 2013). Furthermore, p62 complexes with SQSTM1 could act as a selective autophagy receptor that degrades the autolysosome when autophagic flux increases (Xiang et al., 2021).

Mitochondria, the energy center of cells, are involved in different physiological processes and play essential roles in cancer development and progression. The dysfunction of mitochondria causes various disorders and affects the process of cancer cell survival and death. Accumulating evidence indicates that abnormal mitochondria accelerate tumor development and progression, including proliferation, migration, invasion, oxidative phosphorylation and apoptosis (Fu et al., 2019; Chiu et al., 2020). Although targeting mitochondria is widely recognized as important for effective anticancer therapy, most anti-cancer drugs are difficult to target this organelle (Wu et al., 2018). Based on the differences of mitochondria between normal cells and cancer cells, many mitochondrial-targeted agents have been developed for monitoring drug distribution and achieving specific therapies (Zhang et al., 2011; Zhang et al., 2021).

In particular, researchers have been developing “theranostic” compounds that could not only treat cancers but also detect or monitor their response to therapy. Current theranostics are based mainly on nanotechnology (Vinod and Jena, 2021; Wu et al., 2021) or conjugation with tumor-specific ligands (Zhao et al., 2019), contrast agents (Shu et al., 2021), and anticancer drugs (Kong et al., 2016; Jeyamogan et al., 2021). To some extent, these methods have been proofed to be promising, but there are some debates about the selectivity and specificity of these theranostics (Zhang et al., 2011). Therefore, it is crucial to develop alternative or innovative strategies for solving the above problems. Recently, small molecule-based fluorophores with unique inherent-targeting have attracted interests of researchers, because theranostic agents with this structure not only solves the problem of targeting but also benefits for large-scale application in the future (Wang et al., 2018). For instance, multifunctional heptamethine dyes that can be selectively enriched in the mitochondria of various human tumor xenografts have been developed for tumor imaging and therapy (von Kiedrowski et al., 2020; Zhang et al., 2021). However, they also exert some undesired side effects due to their poor water solubility and non-ideal antitumor activity.

Hemicyanines, as a principal member of the cyanine family, are commonly used in fluorescent sensors owing to their good optical properties, specifically, long absorption and emission wavelength, high fluorescence quantum yield and large Stokes shift (Kong et al., 2016; Zeng et al., 2021). Recently, several studies have reported the use of hemicyanine dyes as fluorescent probes for disease diagnosis in response to various biomarkers, including ROS and nitrogen species (Xie et al., 2016), biothiols (Chen et al., 2015), enzymes (Liu et al., 2019), and pH (Li et al., 2015). Furthermore, hemicyanine dyes have been widely applied for cancer imaging and imaging-guided surgical resection (Luo et al., 2018), but they have rarely been directly used in anti-cancer therapy.

In the present study, we developed the hemicyanine-based fluorescent probe ZWZ-3, which can be selectively enriched in the mitochondria of melanoma cells, thus promoting mitochondrial oxidative phosphorylation and inducing apoptosis and autophagy. In this way, ZWZ-3 shows promise as a novel theranostic agent for characterization and treatment of melanoma.

## Materials and Methods

### Materials

All reactions were performed under magnetic stirring and in dried glassware. Unless otherwise stated, all chemicals and solvents were obtained from commercial sources and used without further purification. Analytical thin-layer chromatography was conducted on 0.20 mm silica gel plates (Haiyang, Qingdao, Shandong, China) under the indicator of QF-254 UV. Column chromatography was performed on Haiyang silica gel 60 (200–300 mesh). Mass spectra was obtained from the Agilent (United States). ^1^H NMR and ^13^C NMR spectra were analyzed on a Bruker DMX600 NMR spectrometer under tetramethyllsilane (TMS) as an internal standard. Peak multiplicity of NMR signals was as stated below: s, singlet; d, doublet; t, triplet; q, quartet; m, multiplet. Chemical shift (δ): ppm relative to Me4Si (internal standard). Coupling constant: J (Hz).

MTT (3-[4,5-dimethyl-2-thiazolyl]-2,5-diphenyl-2-H-tetrazolium bromide, thiazolyl blue tetrazolium bromide), H33342 were purchased from YuanYe Bio-Technology, China. AnnexinV-FITC/PI was purchased from Biosharp, China. DCFH-DA, JC-1, MitoTracker Green were purchased from Beyotime, China. Lipfiter transfection reagent was purchased from Hanbio, China. Sulfobromophthalein (BSP, a competitive inhibitor of OATP transporters) was purchased from BT Reagent, NAC (a pharmacological inhibitor of ROS) was purchased from Bestbio. Antibodies against Atg5 (#12994), P62 (#39749), PARP (#9532T), BAX (#2772S), LC3A/B (#12741S), Ki-67 (#12202S) were purchased from SCT. Cleaved Caspase 9 (#ab202068) was purchased from Abcam. Cleaved Caspase 3 (#341034), β-actin (#T200068-8F10), Anti-mouse HRP (#511203), Anti-rabbit HRP (#511103) were purchased from Zen Bioscience.

### Synthesis and Structural Characterization

A mixture of 2-bromocyclohex-1-ene-1-carbaldehyde **1** (370 mg, 2 mM), 2-hydroxy-5-nitrobenzaldehyde **2** (167 mg, 1 mM) and Cs_2_CO_3_ (1.95 g, 6.0 mM) in DMF (2 ml) was stirred for 12 h at 25°C. The solvent was removed under reduced pressure by evaporation, and the residue was purified by silica gel chromatography to afford 6-nitro-2,3-dihydro-1H-xanthene-4-carbaldehyde **3** as a yellow solid (128 mg, 50% yield). A mixture of 3 (25.7 mg, 0.1 mM), 1,2,3,3-tetramethyl-3H-indol-1-ium **4** (17.4 mg, 0.1 mM) and K_2_CO_3_ (65 mg, 0.2 mM) in Ac_2_O (2.0 ml) was stirred at 80°C for 12 h. The solvent was removed by evaporation under reduced pressure, and the residue was subjected to silica gel chromatography to afford ZWZ-3 as a purple solid (30 mg, 73% yield). ^1^H NMR (400 MHz, CDCl_3_) δ 8.59 (d, 1H, J = 15.32 Hz), 8.01 (d, 1H, J = 15.32 Hz), 7.95 (s, 1H), 7.49–7.53(m, 5H), 7.09(s, 1H), 6.96 (d, 1H, J = 15.32 Hz), 4.25 (s, 3H), 2.85–2.96 (m, 2H), 1.88–1.95 (m, 2H), 1.84 (s, 6H), MS: m/z calculated for C_26_H_25_N_2_O_3_^+^, 413.1860, found 413.1860.

### Determination of Optical Properties

To study the optical properties of ZWZ-3 in methanol, serum and phosphate buffered saline (PBS), ZWZ-3 was incubated with methanol or 10% fetal bovine serum (FBS, Gibco) or PBS at 37°C. The absorbance of ZWZ-3 was measured on a UV–Vis scanning spectrophotometer Puxi TU-1900. Fluorescence intensities were detected using an Agilent F-7000 fluorescence spectrophotometer (United States) (550 nm excitation, continuous wavelength from 500 to 600 nm emission).

### Cell Lines and Cell Culture

Murine melanoma cell line (B16), human melanoma cell line (A375) and murine macrophage cell line (RAW264.7) were obtained from ATCC (Manassas, VA, United States) and cultured in ATCC suggested media with 10% FBS. Cells were supplied with 1% antibiotic-antimycotic solution and incubated at 37°C humidfied atmosphere with 5% CO_2_.

### MTT and Colony Formation Assays

The effect of ZWZ-3 on cells viabilities was performed by standard MTT method and colony formation assay. In the MTT assay, the cells were seeded in 96-well plates at 1–5×10^3^ cells/well and incubation for 24 h. 100 μl medium with various concentrations of ZWZ-3 were added to each well and incubated for 24, 48 and 72 h, respectively. Then, 20 μl MTT solution were added to each well to a final concentration of 0.5 mg/ml, followed by incubation for 2–4 h at 37°C. Finally, the medium of each well was replaced by 150 μl DMSO and incubated for 15–20 min. The absorbance of each well was detected at 570 nm wavelength using Spectra MAXM5 microplate spectrophotometer (Molecular Devices), then the growth inhibiting rates was calculated. Data were derived from at least three separate experiments.

In the colony formation assay, the cells were seeded in six-well plates at 200–500 cells/well and incubated for 24 h. Then the cells were incubated with 2 ml medium containing indicated concentrations of ZWZ-3 for 7 days. After washing with PBS, fixed with 4% paraformldehyde and stained with crystal violet solution (0.5% in methanol), the cells were observed and counted by the microscope. Each assay was replicated three times.

### Detection of ΔΨm and ROS Levels in Cells

DCFH-DA (6.7 μM) and JC-1 (10 μg/ml) were used to measure mitochondrial membrane potential (ΔΨm) and reactive oxygen species (ROS) levels. After treated with ZWZ-3 for 2 h or 24 h, the melanoma cells were treated with DCFH-DA or JC-1 at 37°C in the dark for 40 or 20 min. The cells were washed with PBS, and the fluorescence of cells was observed using fluorescence microscope. Image J software was employed to measure the fluorescence intensity. Each assay was replicated three times.

### Fluorescent Imaging For Biodistribution and Tumor-Targeted

Confocal laser technique was conducted to assess for subcellular colocalization of ZWZ-3 *in vitro*. In brief, A375, B16 and RAW264.7 cells in the logarithmic growth phase were seeded in 35 mm glass-bottomed culture dishes at 37°C and 5% CO_2_ for 24 h. After co-incubating with ZWZ-3 (5 μM) for 1 h, the fresh medium was replaced. Then, apply MitoTracker Green (Beyotime, China) to make the final concentration of 200 nM, incubated for 45 min. After washed with PBS for three times, the cells were observed by laser scanning confocal microscope (Leica TSC SP8) (ZWZ-3: 638 nm excitation, continuous wavelength from 450 to 700 nm emission; MitoTracker Green: 490 nm excitation, continuous wavelength from 450 to 700 nm emission).

To assess the localization of ZWZ-3 *in vivo*, the organs of subcutaneous tumor model mice were made into frozen sections for observation. Briefly, after the tissues of heart, liver, spleen, lung, kidney, paracancerous and tumor were cut into frozen sections on a cryostat microtome (Leica CM1950), they were fixed with ice acetone for 15 min, drained and washed with PBS for 5 min. Then, the frozen sections were stained by Hoechst33342 (2 μg/ml) (YuanYe Bio-Technology, Shanghai, China) at 37°C for 10 min and washed with PBS for two to three times, 5 min each time. A fluorescence microscope (Leica DMi8) was performed for observing.

To investigate the mechanism affecting the selective entry of ZWZ-3 into tumor cells, B16 cells were added in six-well plates and cultured overnight. B16 cells were incubated with 250 µM BSP for 20 min and then incubated with 5 µM ZWZ-3 for 1 h. After being washed with PBS solution, cells were incubated with 1 μg/ml Hoechst33342 for 10 min. Finally, the cells were cleaned by PBS solution and observed by fluorescence microscope.

### Detection of Cells Apoptosis

The detection of cells apoptosis was conducted according to Annexin V-FITC/PI detection kit (Biosharp, China). The A375 and B16 cells (2 × 10^5^ cells/well) in the logarithmic growth phase were seeded in six-well plates and incubated overnight. After treatment with various concentration of ZWZ-3 (0 ∼ 2.5 μM) for 24 h, the cells were collected, cleaned with cold PBS and stained with 5 μl Annexin V-FITC at room temperature in the dark for 20 min. Subsequently, 10 μl of PI was added for 5 min. FCM was used to measure the apoptosis induced by ZWZ-3. The data was carried out using FlowJo software.

### Western Blotting Analysis

The B16 cells were collected, lysed and quantified (BCA; Beyotime, China). Western blot analyses were conducted using the indicated antibodies. Protein densitometric analysis of all bands were performed using the Image J software. Data were depicted as fold difference over untreated control. GAPDH was conducted as an internal control.

### Measurement of GFP-LC3 Aggregates

For visualization of the autophagosomes, cells were transfected with GFP-LC3 plasmid, which was presented to me by Dr. F. Jianguo (Southwest Medical University, Luzhou, China). Briefly, B16 cells were seeded into a 24-well plate and cultured overnight. Next, cells were transfected with GFP-LC3 plasmid for 12 h. The cells were exposed to different concentration of ZWZ-3 for 12 or 24 h. Lastly, fluorescence microscope was conducted to observe the GFP-LC3 aggregates.

### Tumor Xenograft Models

Mice used in this study were purchased from the Chengdu Dossy Laboratory Animals Company (Chengdu, China) and were housed in a specific-pathogen-free (SPF) condition facility with an air-conditioned room at 25 ± 2°C with 40%–70% relative humidity, and a 12-h light/dark cycle. For subcutaneous tumor model, mice engrafted subcutaneously with 1 × 10^7^ A375 cells were randomized to groups when tumor volume was around 100 mm^3^ and were given by intraperitoneal injection of ZWZ-3 5 mg/kg or vehicle once 3 days. Tumor size and body weight were measured every 3 days. The mice were sacrificed when reached the endpoint defined by the tumor size (∼1,000 mm^3^). Tumor volume is calculated as follows: Volume = 0.5 × a × b^2^, where a (mm) represents the length and b (mm) represents the width of the tumor. All animal experiments have been approved by the Laboratory Animal Management Committee of the Affiliated 190 Hospital of Southwest Medical University in China (Permit Number: 20200201) and were conducted in accordance with the approved guidelines.

### Hematoxylin and Eosin (H&E) Staining

The tissues of mice were fixed with 10% formaldehyde solution for 24 h and embedded in paraffin, then cut into 4 mm-thick sections. Finally, the sections were stained by hematoxylin and eosin (H&E) and observed using microscope (Nikon, NiE, Japan).

### Immunohistochemistry

Immunohistochemical staining for Ki-67 and CC-3 was performed on xenograft tumor tissues using antibodies against Ki-67 and CC-3 respectively.

### Statistical Analysis

The data were expressed as the mean ± standard deviation and analyzed using SPSS 17.0 software. The differences of two groups were analyzed by *t*-test of two independent samples. *p* < 0.05 were considered statistically significant.

## Results

### Chemical Synthesis and Optical Properties of ZWZ-3

**FIGURE 1:**
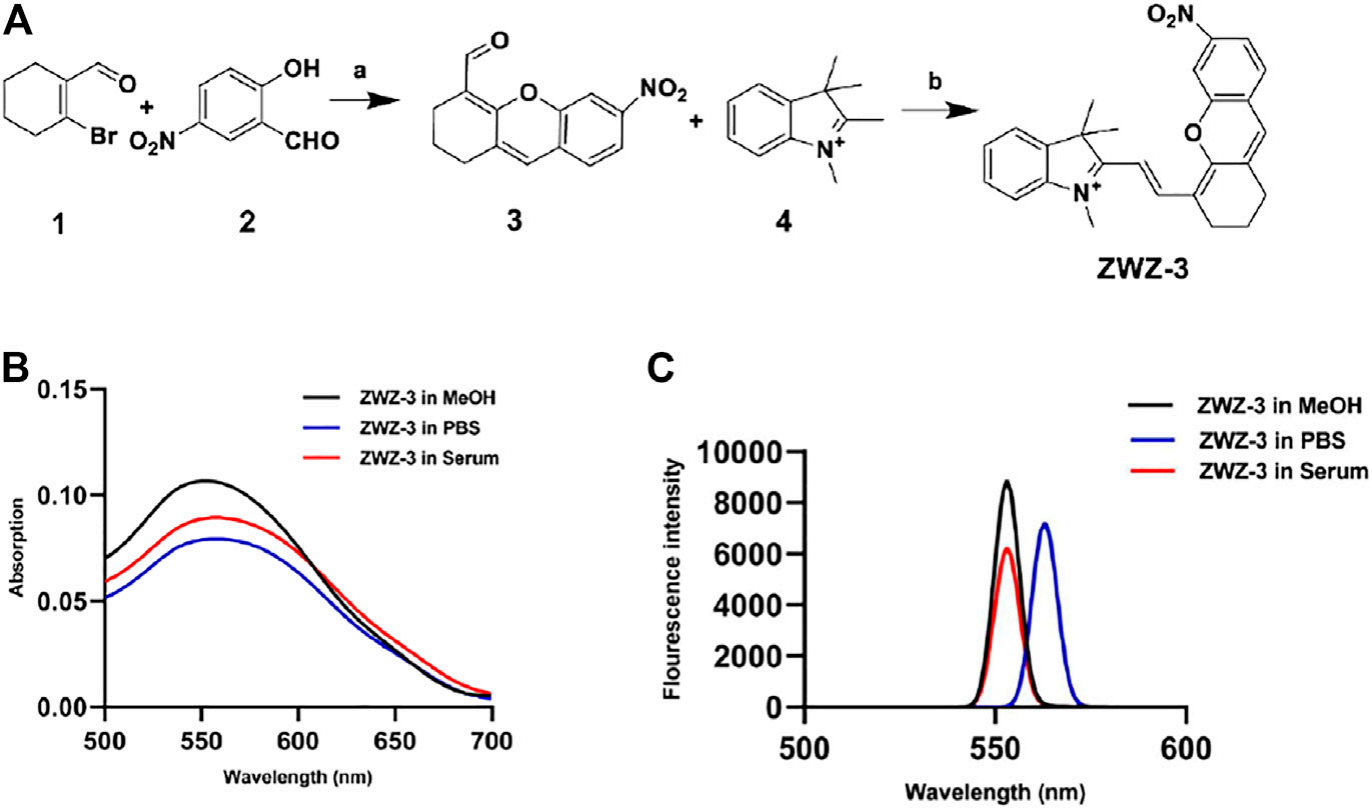
Chemical synthesis and optical properties of ZWZ-3. **(A)** Synthetic route and chemical structure of ZWZ-3. a: Cs_2_CO_3_, DMF, 25°C, 12 h, yield: 50%; b: K_2_CO_3_, Ac_2_O, 80°C, 12 h, yield: 73%. **(B)** The absorption spectra of 5 μM ZWZ-3 in methanol, PBS and 10% foetal bovine serum (FBS), respectively. **(C)** The fluorescence intensity of 5 μM ZWZ-3 in methanol, PBS and 10% FBS, respectively. All experiments were performed in triplicate.

The general synthetic route of compound ZWZ-3 is illustrated in Figure 1A. The structural characterization of ZWZ-3 was determined by ^1^H-NMR (Supplementary Figure S1), 13C-NMR (Supplementary Figure S2) and high resolution mass spectrometry (HRMS) (Supplementary Figure S3). The absorption and fluorescence spectra of ZWZ-3 were investigated in methanol (MeOH), PBS and 10% fetal bovine serum. It was found that the absorption and emission peak of ZWZ-3 was in the 550–560 nm (Figure 1B). ZWZ-3 presented a good stability in 10% FBS and methanol (Figure 1C), indicating that it has potential as a novel fluorescence probe for biomedical imaging.

### ZWZ-3 Specifically Localizes to the Mitochondria of Cancer Cells and Targets Tumor Tissues

**FIGURE 2:**
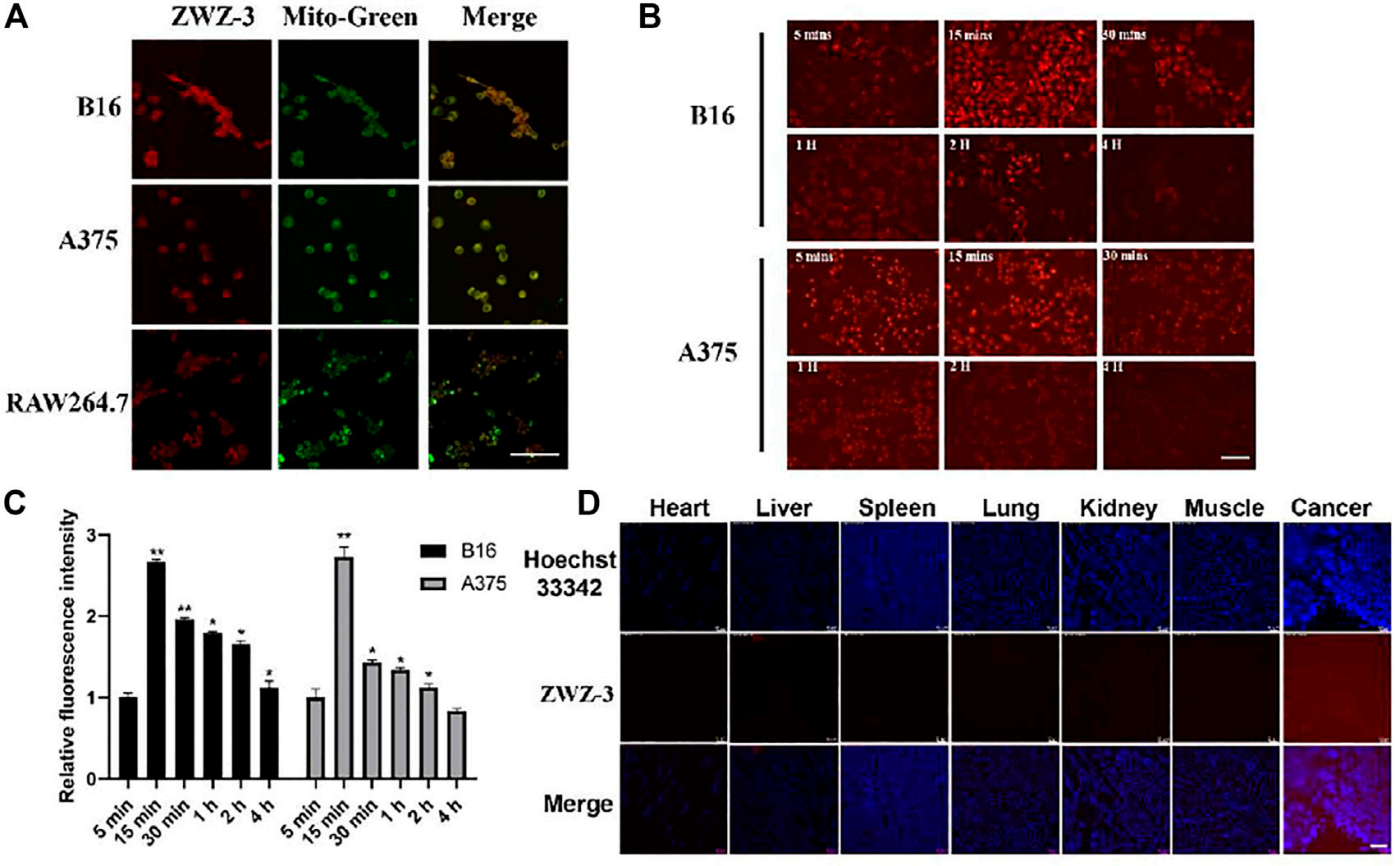
ZWZ-3 specifically locates to the mitochondria of cancer cells and targets tumor tissue. **(A)** Colocalization of ZWZ-3 with a mitochondria-specific tracker (Mito Tracker Green) in B16, A375 and RAW264.7 cells, imaged using a confocal microscope. **(B) (C)** fluorescence intensity in B16 and A375 cells was evaluated after incubation with the same concentration of ZWZ-3 (5 μM) for various times (*n* = 3). **(D)** C57BL/6J mice with B16 subcutaneous tumor xenografts were subjected to histopathologic analysis after a single-dose intravenous administration of ZWZ-3 at 2 mg/kg for 24 h. Organs and xenografts were imaged by fluorescence microscope. All experiments were conducted in three time and each point represents the mean ± SD (*p < .05; **p < .01). Scale bar = 100 µm.

Hemicyanines are known to target mitochondria due to the presence of cationic nitrogen (Li et al., 2016). A subcellular localization assay, using colocalization with a mitochondrial tracker (MitoTracker Green) in B16, A375 and RAW264.7 cells, showed that ZWZ-3 preferentially accumulated in the mitochondria of tumor cells in a time-dependent manner (Figures 2A–C). Fluorescence began to appear at 5 min, and it was at the strongest at 15 min. After 15 min, the fluorescence intensity gradually weakened and there was almost no fluorescence at 4 h. C57BL/6J mice with B16 subcutaneous tumor xenografts were subjected to histopathologic analysis after a single-dose intravenous administration of ZWZ-3 at 2 mg/kg. Histopathologic analysis of organs and tumor frozen sections (Figure 2D) indicated that ZWZ-3 preferentially accumulates in tumor tissues. Thus, ZWZ-3 could be a promising fluorescence probe for targeting mitochondria and biomedical imaging in melanoma based on our results.

### ZWZ-3 Selectively Accumulates in the Mitochondria of Tumor Cells *via* a Mechanism Dependent on organic anion-transporting polypeptide

**FIGURE 3:**
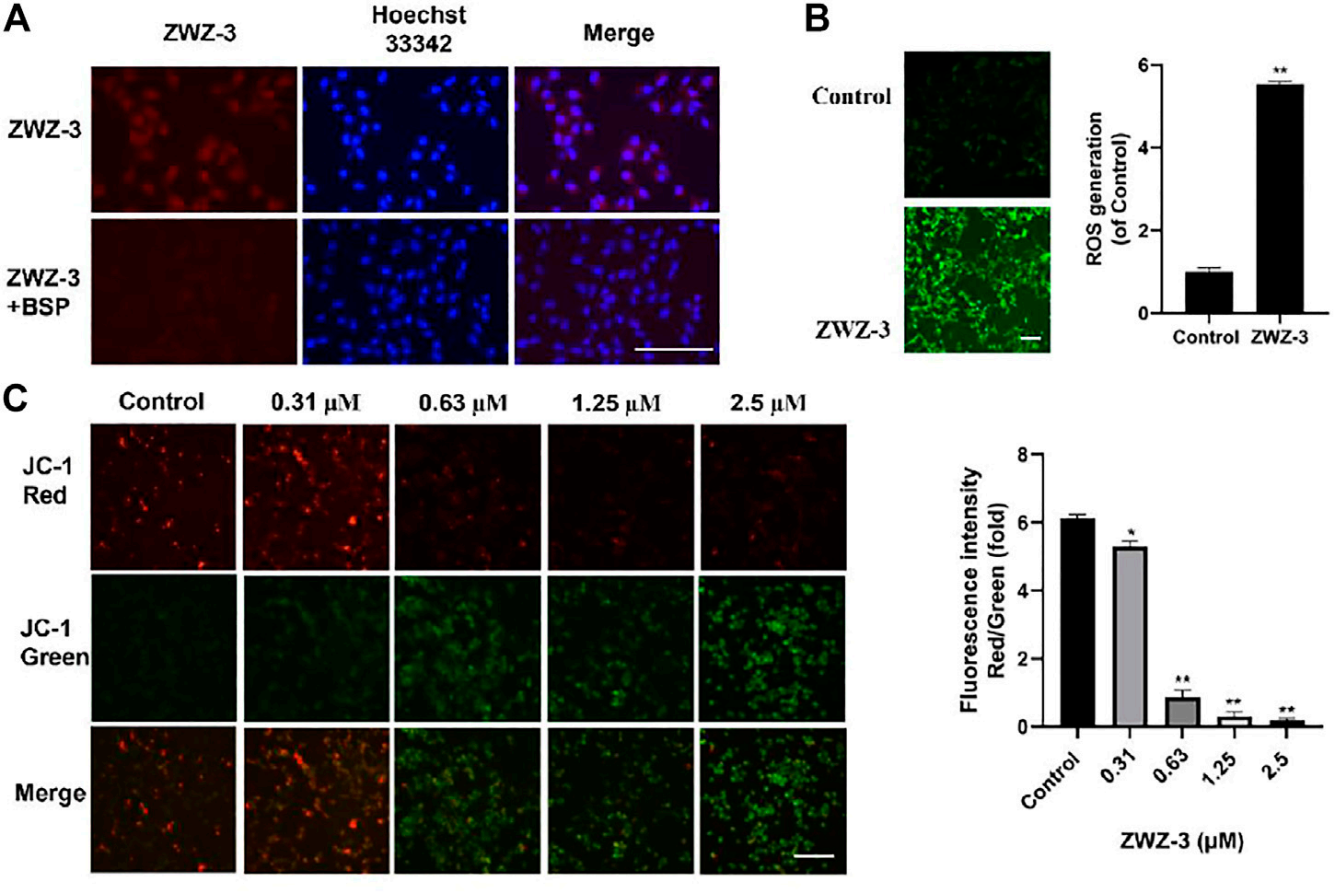
ZWZ-3 enters the mitochondria of tumor cells through OATP to induce the production of ROS and mitochondrial membrane depolarization. **(A)** B16 cells were pretreated with vehicle control, sulfobromophthalein (BSP) (250 μM) for 20 min, then treated with 5 µM ZWZ-3 for 1 h prior to fluorescence microscope observations. **(B)** Intracellular ROS productions were detected using DCFH-DA. **(C)** B16 cells were pretreated with ZWZ-3 for 24 h, then treated with JC-1 for 20 min prior to fluorescence microscope observations. All experiments were carried out in triplicate and each point represents the mean ± SD (*p < .05; **p < .01). Scale bar = 100 µm.

OATPs are multispecific transport proteins, which can transport cations, anions and neutral compounds in cells, and plays an important role in drug absorption, distribution and excretion *in vivo* (Obaidat et al., 2012; Liu et al., 2018). Shi found that OATP mediates the selective accumulation of the heptamethine dye IR-780 in tumor cells (Zhang et al., 2010). To verify whether ZWZ-3 enters tumor cell through the same pathway, B16 cells were pretreated with sulfobromophthalein (BSP, a competitive inhibitor of OATP transporters) for 20 min. BSP significantly inhibited the tumor accumulation of ZWZ-3 (Figure 3A), suggesting that ZWZ-3 enters the cytosol in an OATP-dependent manner. In addition, similar results were observed in A375 cells (Supplementary Figure S4).

Mitochondrial membrane potential (ΔΨm) plays a key role in cell health and function. Mitochondria-mediated intrinsic apoptotic pathway is always accompanied by the disruption of ΔΨm. Here, we treated B16 cells and A375 cells with ZWZ-3 for 24 h, then used the cationic dye (JC-1) to assess ΔΨm. As shown in Figure 3B and Supplementary Figure S5, ZWZ-3 induced mitochondrial membrane depolarization in a dose-dependent manner.

High levels of ROS were shown to promote mitochondria-mediated intrinsic apoptotic pathway (Idelchik et al., 2017). To determine whether ZWZ-3 affects ROS production, ROS products were measured after stimulating the cell with 5 µM ZWZ-3 for 2 h. As shown in Figure 3C, we found that ZWZ-3 increased the intracellular ROS levels in melanoma cells in a dose-dependent manner. These results suggest that the fluorescent probe ZWZ-3 can selectively accumulate in the mitochondria of tumor cells in an OATP-dependent manner, inducing mitochondrial membrane depolarization and ROS production.

### ZWZ-3 Suppresses the Growth of Melanoma Cells *in vitro*

**FIGURE 4:**
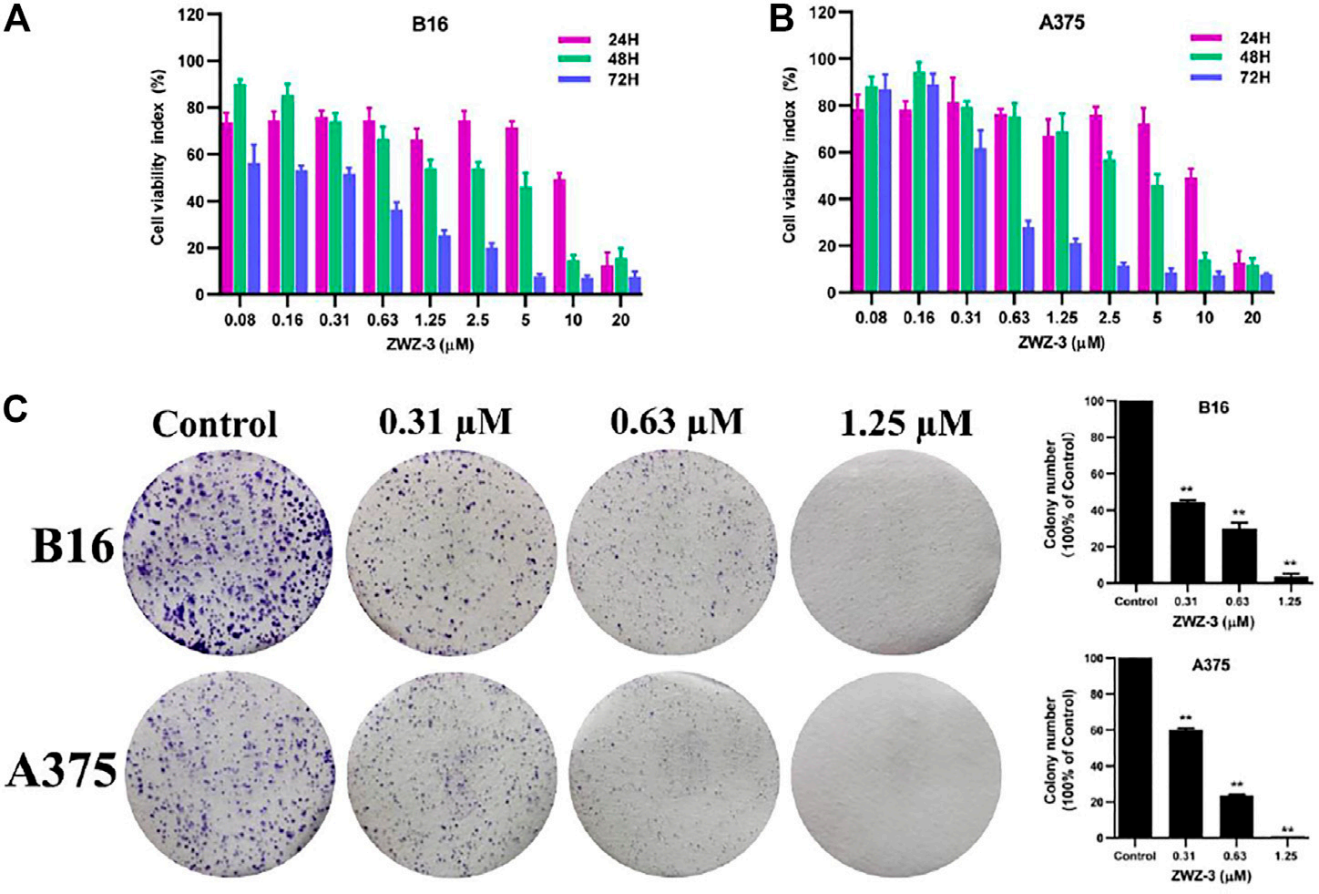
*In vitro* anticancer activity of ZWZ-3 against melanoma cells. **(A,B)** melanoma cell lines (B16 and A375) were treated with various concentrations of ZWZ-3 for 24, 48 and 72 h respectively, and IC_50_ values were calculated. **(C)** B16 and A375 cells were treated with control or gradient concentrations of ZWZ-3 for 7 days. Then, the colony formation in each group was counted. Quantification is displayed on the right of each cell line. Dates are expressed as mean ± SD for 3 independent experiments (**p < .01).

Given the tumor targeting and imaging property of ZWZ-3, further investigation of anti-tumor effects of ZWZ-3 was conducted. The effect of ZWZ-3 on cancer cells proliferation was evaluated by MTT assay (Supplementary Figure S6). In A549 and MDA-MB-231 cell lines, the IC_50_ of ZWZ-3 were calculated as 0.48 and 0.93 μM, respectively. ZWZ-3 displayed relatively strong activity against B16 and A375 cell lines (IC_50_ 0.2 and 0.43 μM, respectively) at 72 h. These results indicated that among the tested tumor cell lines, melanoma cells were the most sensitive to ZWZ-3. Additionally, ZWZ-3 suppressed melanoma cells viability in a concentration and time—dependent manner (Figures 4A,B). Similarly, in a colony formation assay, ZWZ-3 suppressed proliferation of both cell lines and reduced colony numbers in a dose-dependent manner (Figure 4C).

### ZWZ-3 Induces Autophagy by Regulating Autophagy-Related Proteins in Melanoma Cells

**FIGURE 5:**
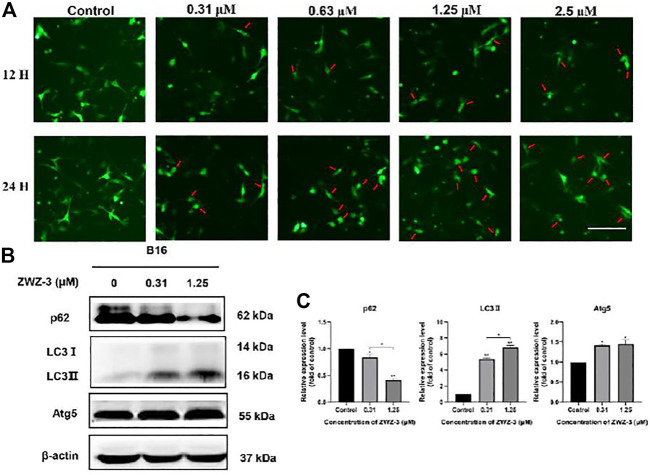
ZWZ-3 promotes autophagy in melanoma cells. **(A)** B16 cells expressing GFP-LC3 were treated with complete medium or ZWZ-3. The distribution of GFP-LC3 was examined using fluorescence microscope. **(B,C)** The levels of P62, LC3-II and Atg5 were determined via western blotting. Protein expressions were qualified by the densitometry analysis using ImageJ. Dates are expressed as mean ± SD for 3 independent experiments (*p < .05, **p < .01).

The role of autophagy in cell survival and death has been reported in several studies. Accumulating evidence has revealed that LC3 (microtubule-associated protein 1A/1B-light chain 3) is a vital autophagosome marker and that p62/SQSTM1 is a selective autophagy receptor that is degraded within the autolysosome after an increase in autophagic flux (Xiang et al., 2021). Therefore, to detect the effect of ZWZ-3 on autophagy, we evaluated the level of crucial autophagic marker LC3-II *via* B16 cells that expressed GFP-LC3. We found that ZWZ-3 could efficiently induce GFP-LC3 puncta in a dose- and time-dependent manner (Figure 5A). Additionally, as shown in Figures 5B,C, p62 protein levels were notably decreased after ZWZ-3 treatment. LC3-II and Atg5 protein levels were increased following ZWZ-3 treatment for 24 h in B16 cells. The above-mentioned results indicated that ZWZ-3 induced autophagy in a concentration- and time-dependent manner in melanoma cells.

### ZWZ-3 Induces Mitochondria-Mediated Apoptosis in Melanoma Cells

**FIGURE 6:**
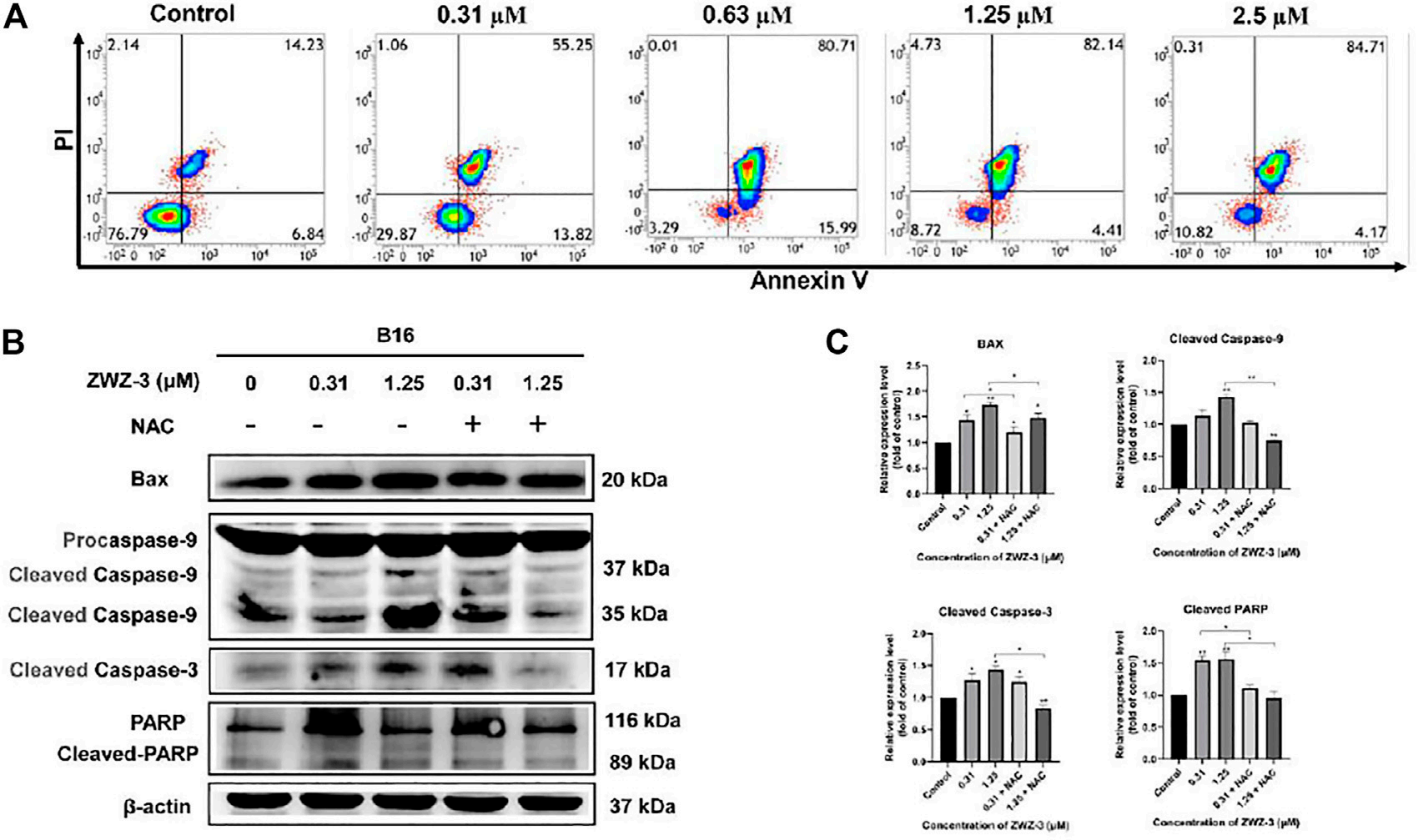
ZWZ-3 induces apoptosis in melanoma cells. **(A)** FCM analysis of B16 cells stained with Annexin V-FITC/PI after treatment with ZWZ-3 for 24 h **(B,C)** Cells were pretreated with ROS inhibitors (NAC, 10 mM) for 30 min followed by treatment with ZWZ-3 for 24 h. The expression of Bax, cleaved caspase 9, cleaved caspase 3 and cleaved PARP were measured by western blotting. Protein expressions were qualified by the densitometry analysis using ImageJ. Dates are expressed as mean ± SD for 3 independent experiments (*p < .05, **p < .01).

To explore whether ZWZ-3 induce apoptosis in melanoma cells, we treated B16 cells or A375 cells (Supplementary Figure S7) with different ZWZ-3 concentrations, then analyzed them by flow cytometry. Treatment with 0.31 μM ZWZ-3 for 24 h led to an apoptosis rate of 55.25% (Figure 6A), and this effect was dose-dependent, since 0.63 μM led to a rate of 80.71%; 1.25 μM, 82.14%; and 2.5 μM, 84.71%. These data indicated that ZWZ-3 induced melanoma cell apoptosis in a dose-dependent manner. Western blot analysis displayed that ZWZ-3 increased the levels of Bax, cleaved caspase 9, cleaved caspase 3 and cleaved PARP (Figures 6B,C). These effects of ZWZ-3 were reversed by NAC (a pharmacological inhibitor of ROS), suggesting that the fluorescent probe ZWZ-3 induces mitochondria-mediated apoptosis through the ROS pathway.

### ZWZ-3 Inhibits Melanoma Growth *in vivo*

**FIGURE 7:**
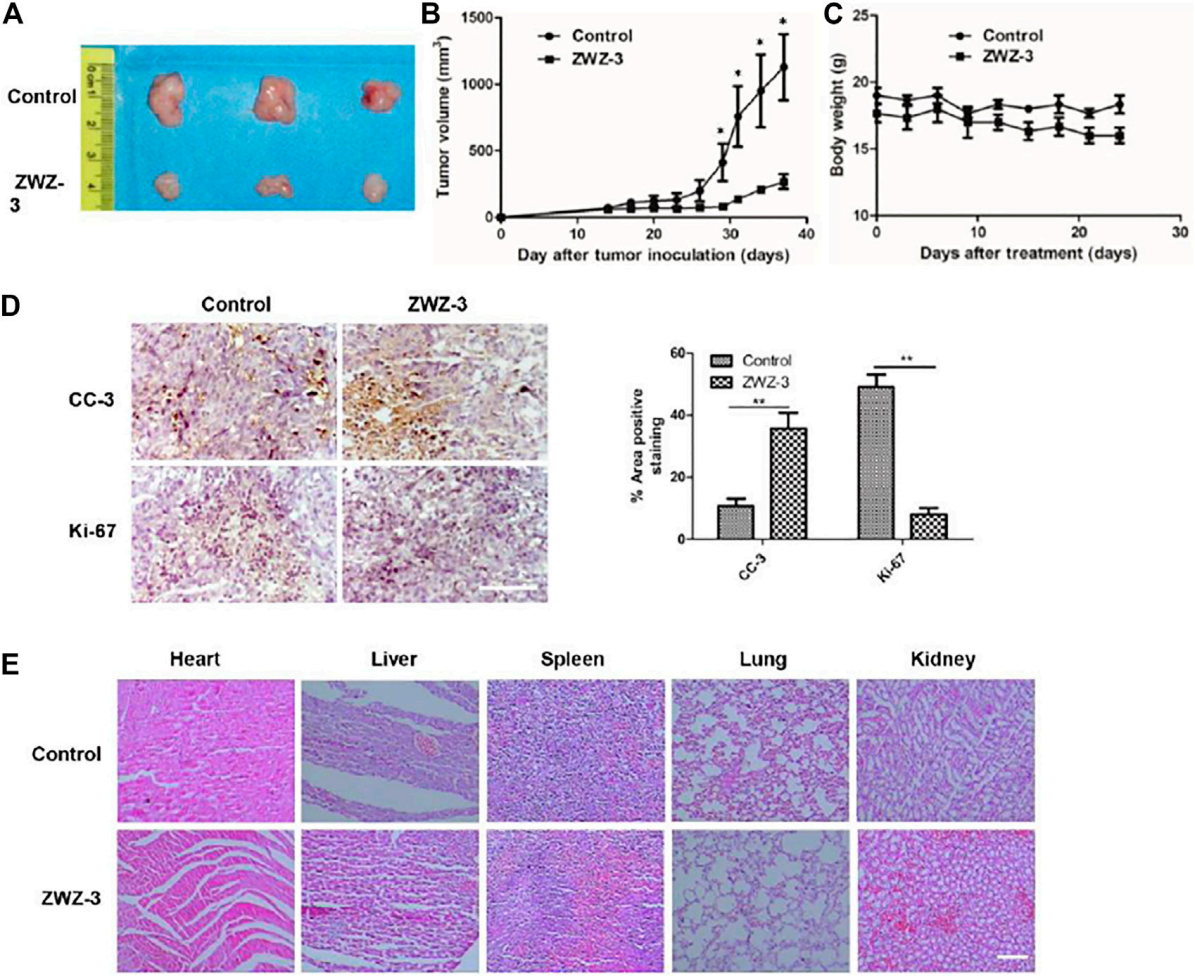
ZWZ-3 reduces tumor cell proliferation and induces tumor apoptosis *in vivo*. **(A)** Tumor pictures from mice treated with indicated ZWZ-3 on the final day. In A375 xenograft model, the mice were treated with ZWZ-3 (5 mg/kg) or vehicle. Tumor volumes **(B)** and body weight **(C)** were measured every 3 days. **(D)** Tumor tissues from A375 xenograft treated with control or ZWZ-3 were immunohistochemically analyzed with anti-Ki67 and cleaved caspase 3 (CC-3) antibodies. The statistical data of CC-3 and Ki-67 positive cell number were displayed on the right. **(E)** H&E stain vital organ sections of mice treated with vehicle or ZWZ-3. Dates are expressed as mean ± SD for 3 independent experiments (*p < .05; **p < .01). Scale bar = 100 µm.

To evaluate the potential therapeutic effect of ZWZ-3 *in vivo*, A375 cells were injected into the right flank of BALB/c nude mice. ZWZ-3-treated mice show significantly reduced tumor volume compared to the control group, without significant body weight loss (Figures 7A–C). The growth inhibition rates at day 38 post-inoculation were 76.3%.

Tumor tissues form untreated and ZWZ-3-treated mice were immunohistochemically analyzed to investigate the potential mechanisms through which ZWZ-3 inhibited A375 melanoma growth. Compared with the control group, ZWZ-3 significantly reduced the number of Ki67-positive cells and increased the number of cleaved caspase-3-positive cells (Figure 7D). These results suggest that ZWZ-3 can inhibit proliferation and promote apoptosis in melanoma tumors, consistent with our *in vitro* experiments. Moreover, H&E staining of vital organs (Heart, liver, spleen, lung, kidney) showed that long-term administration of ZWZ-3 did not affect tissue architecture or cell morphology (Figure 7E). These data indicated that ZWZ-3 significantly inhibited melanoma growth without causing obvious toxicities *in vivo*.

## Discussion

Melanoma is a highly aggressive cancer that accounts for most deaths related to skin cancer, yet it is usually diagnosed at late stage and its prognosis is poor. In the present study, we synthesized and identified a new tumor-targeted fluorophore dye ZWZ-3 with great optical properties and stability in 10% FBS and methanol, and tselectively accumulates in the mitochondria of melanoma cells. In a mouse model, ZWZ-3 preferably accumulated in tumor tissues within 24 h after intravenous injection, and it suppressed tumor growth without obvious side effects. These results justify for further investigation of ZWZ-3 as a safe and effective theranostic agent for imaging and treatment of melanoma.

As a cationic lipophile, the heptamethine core can specifically accumulates in the mitochondria of cancer cells through an OATP-mediated pathway (Wang et al., 2018). Consistently, our hemicyanine-based fluorescent probe ZWZ-3 selectively accumulated in the mitochondria of melanoma cells in an (OATP)-dependent manner, inducing mitochondrial ROS production and promoting cell apoptosis in a time- and dose-dependent manner. In addition, ZWZ-3 significantly reduced ΔΨm, while upregulating Bax and activating caspase-3, caspase-9, and PARP. These effects of ZWZ-3 were reversed by adding NAC to inhibit ROS production, suggesting that ZWZ-3 induces apoptosis by increasing ROS levels (Yang et al., 1998).

Autophagy helps normal cells resist nutrient deprivation or metabolic stress (Chen et al., 2021), and it can be exploited for anticancer therapy. Radiation and chemotherapy work, in part, by inducing autophagy in cancer cells (Amaravadi et al., 2019; Li et al., 2020). Two hallmarks of autophagy are the conversion of the soluble form of LC3-I to the autophagosome-associated form LC3-II, as well as the degradation of p62 (Zheng et al., 2018). ATG5 helps catalyze the LC3 lipidation, which is essential for autophagosome formation and expansion (Yang et al., 2021). In our study, ZWZ-3 significantly increased the levels of ATG5 and LC3-II in a dose-dependent manner while promoting degradation of p62, indicating that ZWZ-3 can effectively induce autophagy by regulating the expression of autophagy-related proteins.

Our *in vivo* studies in mice bearing A375 xenografts showed that ZWZ-3 effectively suppressed tumor growth at an inhibitory rate of 76.3%. Consistent with *in vitro* results, tumor sections of ZWZ-3-treated mice showed significantly upregulated cleaved caspase-3 (indicating apoptosis), and downregulated Ki-67 (indicating reduced proliferation). At the same time, ZWZ-3 showed no obvious toxicity *in vivo*, suggesting its potential as a safe, effective theranostic agent.

In summary, we designed and synthesized a novel theranostic agent that can selectively accumulate in the mitochondria of melanoma cells in an OATP-dependent manner. ZWZ-3 can inhibit proliferation and induce apoptosis of melanoma cells by altering levels of various apoptosis- and autophagy-related proteins. Our work may promote the development of this and other dual-functional hemicyanine-based dyes as novel tumor-targeting therapeutic agent.

## Data Availability

The original contributions presented in the study are included in the article/Supplementary Material, further inquiries can be directed to the corresponding authors.
