# Supplementary material for: ZWZ-3, a Fluorescent Probe Targeting Mitochondria for Melanoma Imaging and Therapy
Source: Front Pharmacol. 2022 Feb 23;13:829684. doi: 10.3389/fphar.2022.829684 (PMC8905922; doi:10.3389/fphar.2022.829684)
Supplement: Supplementary file 1 [file DataSheet1.docx]

**ZWZ-3, a Fluorescent Probe** **Targeting Mitochondria for Melanoma Imaging and Therapy**

**Zengjin Liu^1†^, Hailan Wang^2,3†^, Changzhen Sun^1^, Yuanmin He^2^, Tong Xia^2^, Jianv Wang^2^, Xia Xiong^2^, Qingbi Zhang^3^, Sijin Yang^1#^, Li Liu^2#^.**

^1^National Traditional Chinese Medicine Clinical Research Base and Drug Research Center of the Affiliated Traditional Chinese Medicine Hospital of Southwest Medical University, Luzhou 646000, China.

^2^Department of Dermatology, The Afﬁliated Hospital of Southwest Medical University, Luzhou 646000, China

^3^School of Public Health, Southwest Medical University, Luzhou 646000, China


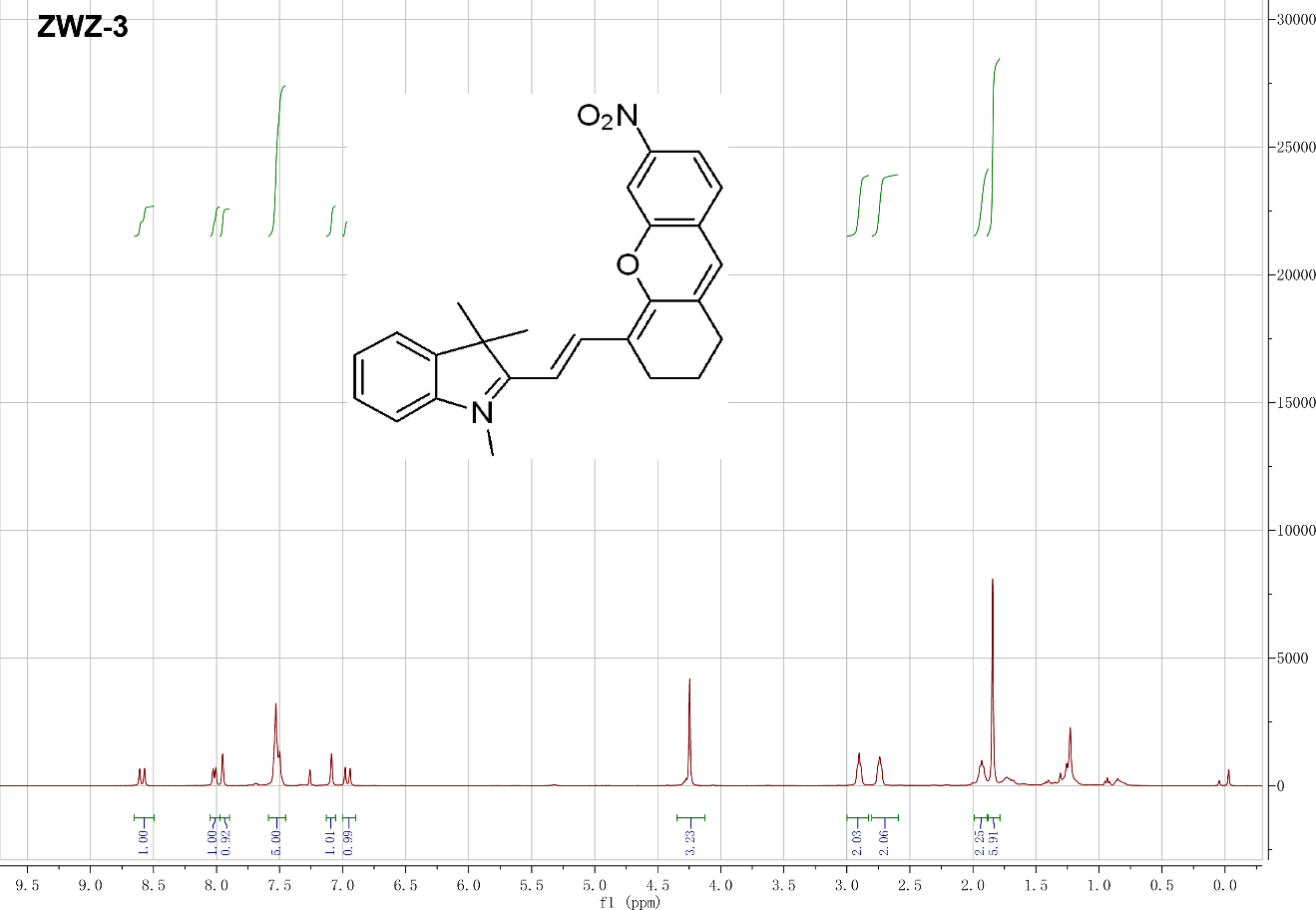


Supplementary Figure 1. ^1^H-NMR of ZWZ-3.


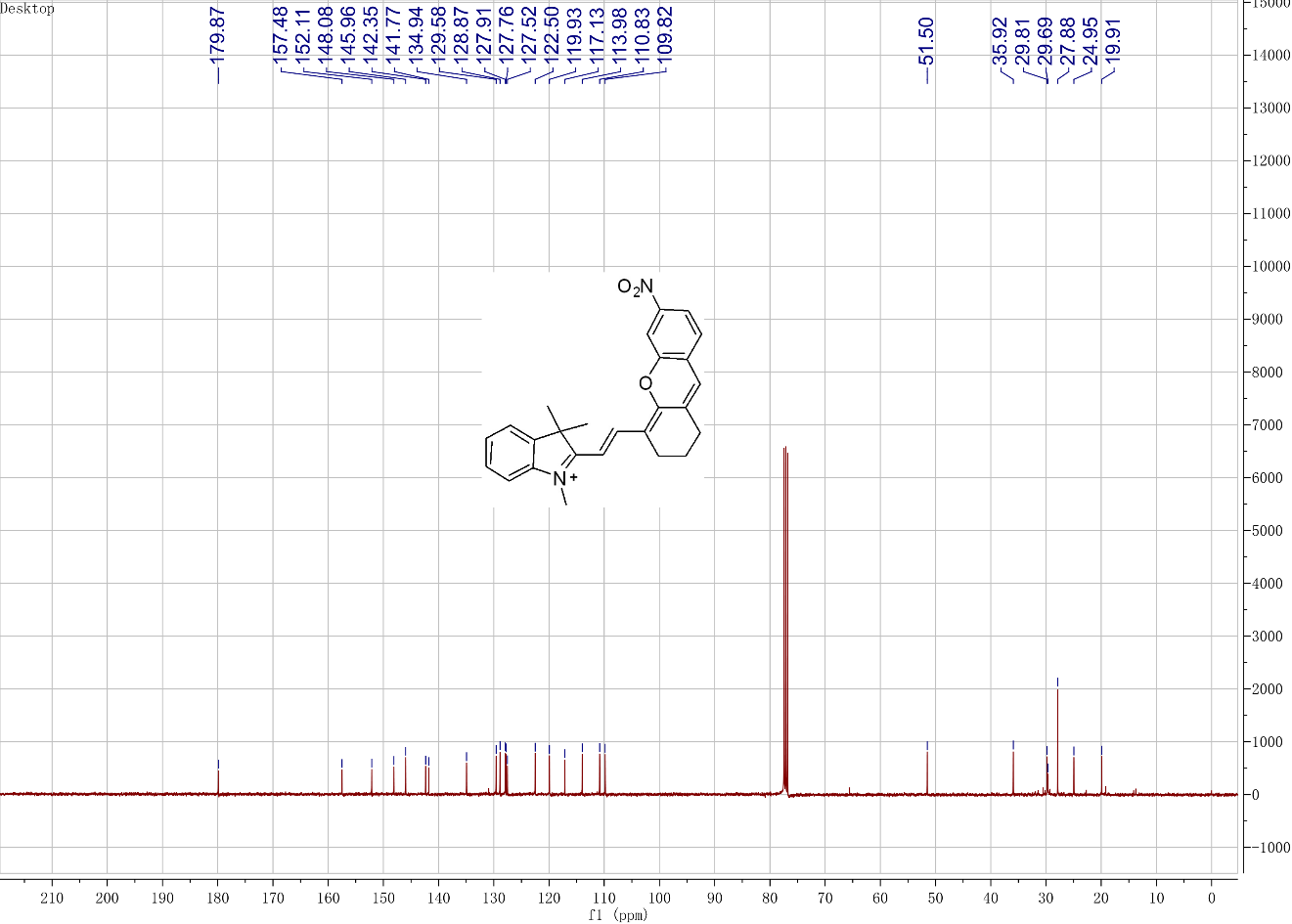


Supplementary Figure 2. ^13^C-NMR of ZWZ-3


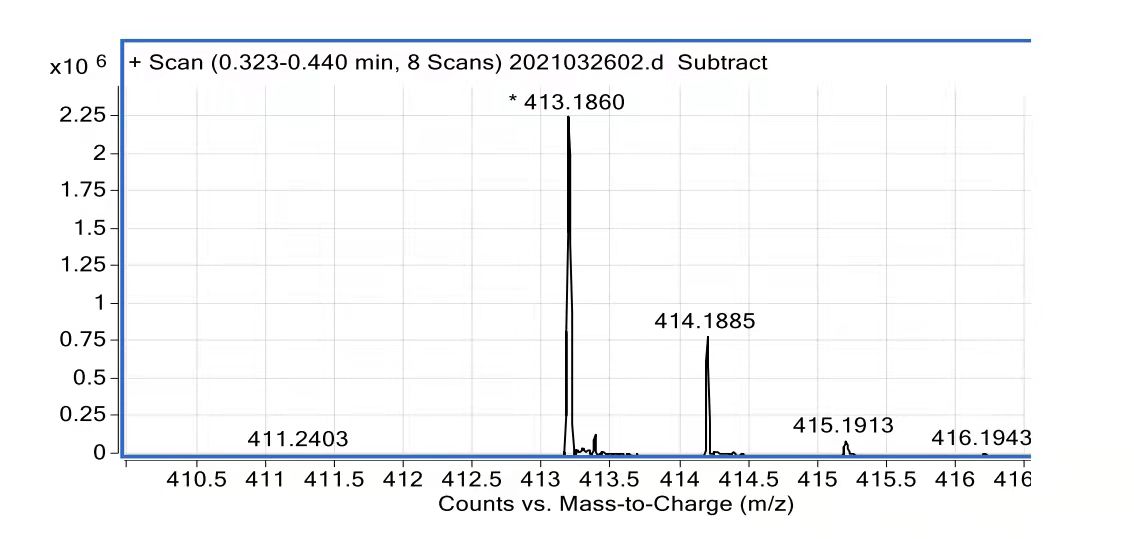


Supplementary Figure 3. HRMS of ZWZ-3


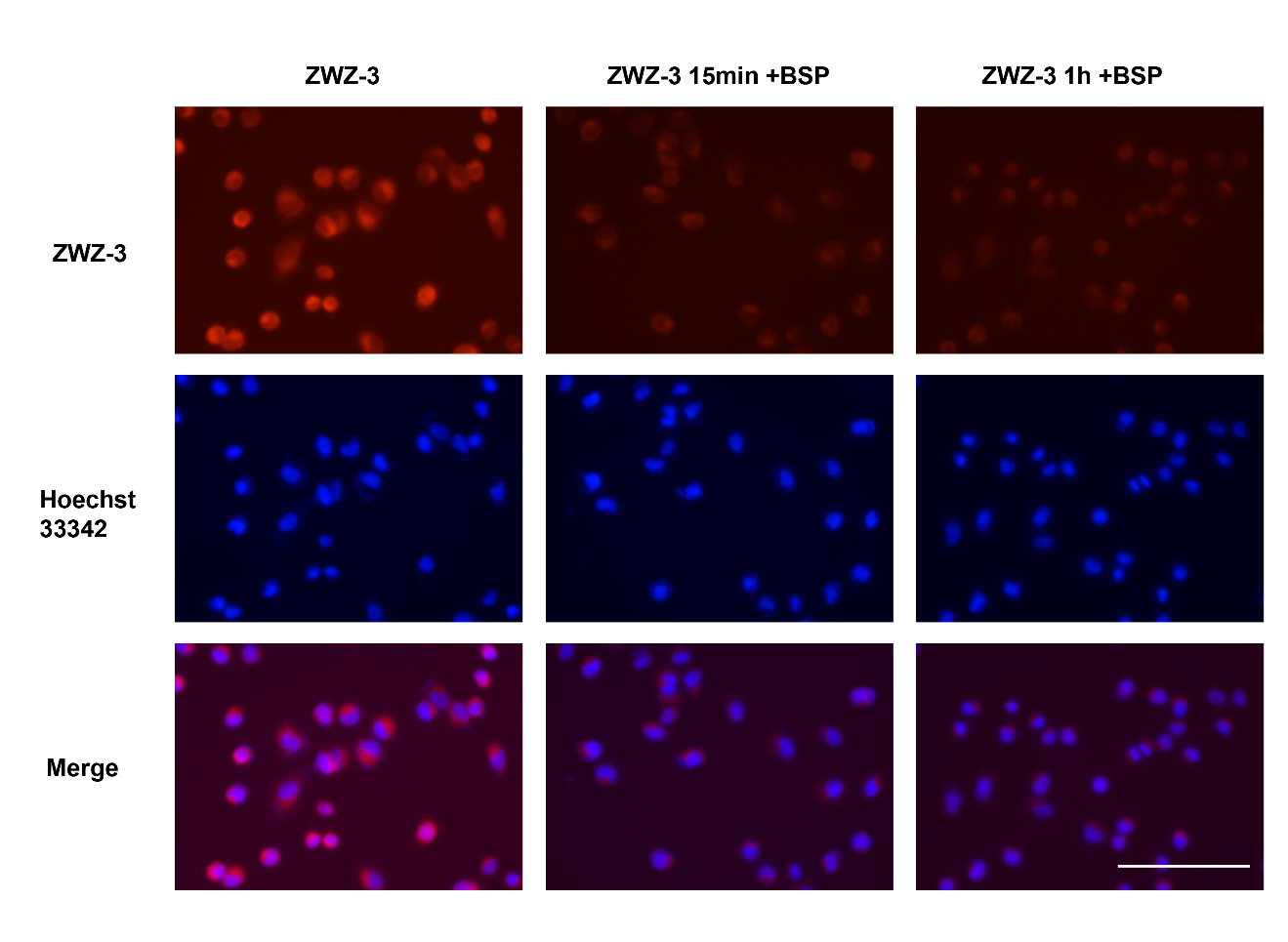
Supplementary Figure 4. ZWZ-3 enters the mitochondria of A375 cells through OATP. A375 cells were pretreated with vehicle control, sulfobromophthalein (BSP) (250 μM) for 20 min, then treated with 5 µM ZWZ-3 for 15 min or 1h prior to fluorescence microscope observations. Scale bar = 100 µm.


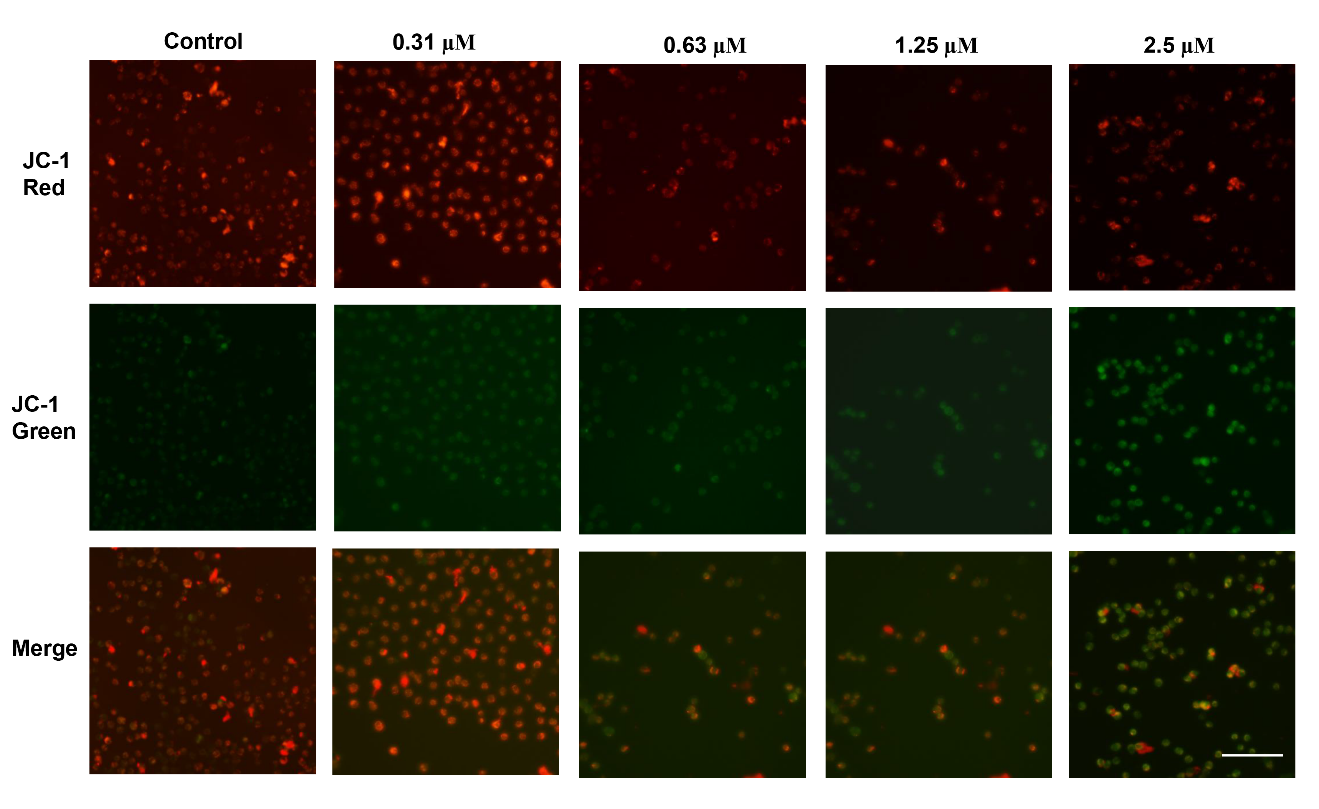
Supplementary Figure 5. ZWZ-3 induces mitochondrial membrane depolarization. A735 cells were pretreated with ZWZ-3 for 24 h, then treated with JC-1 for 15 min prior to fluorescence microscope observations. Scale bar = 100 µm.


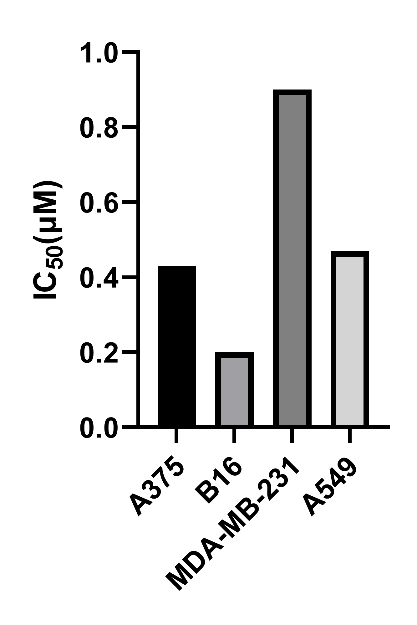


Supplementary Figure 6. The proliferation inhibitory effect of ZWZ-3 against cancer cell lines for 72 h.


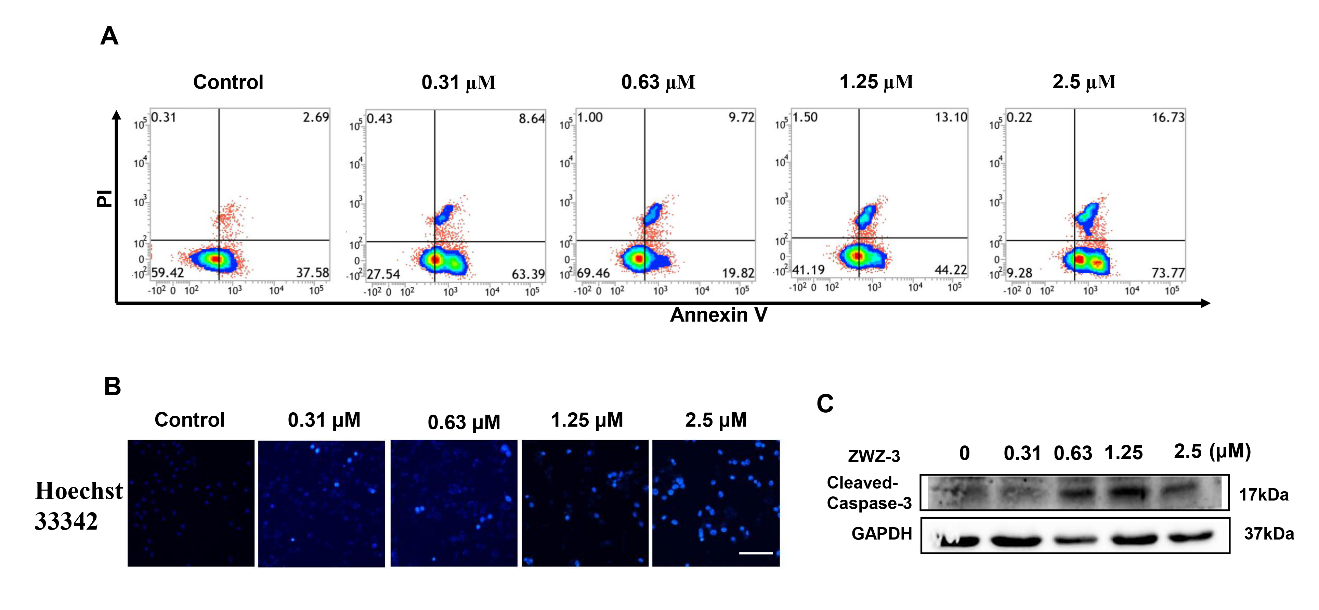


Supplementary Figure 7. ZWZ-3 induces apoptosis in melanoma cells. (A) FCM analysis of A375 cells stained with Annexin V-FITC/PI after treatment with ZWZ-3 for 24 h. (B) Cells were pretreated with ZWZ-3 for 24 h and stained with Hoechst 33342. (C) Cells were pretreated with ZWZ-3 for 24 h. The expression of cleaved caspase 3 was measured by western blotting. Scale bar = 100 µm.
